# Biomechanical properties of masticatory balance in cases with RPDs—The influence of preferred and nonpreferred chewing side: A pilot study

**DOI:** 10.1002/cre2.576

**Published:** 2022-04-28

**Authors:** Lydia Eberhard, Stefan Rues, Lea Bach, Jürgen Lenz, Hans J. Schindler

**Affiliations:** ^1^ Department of Prosthodontics University of Heidelberg Heidelberg Germany; ^2^ Institute of Mechanics Karlsruhe Institute of Technology Karlsruhe Germany; ^3^ Department of Prosthodontics University of Würzburg Würzburg Germany

**Keywords:** bite force, chewing performance, EMG, RPD, X_50_ value

## Abstract

**Objectives:**

Removable partial dentures (RPDs) are inserted with the aim to restore masticatory function. There is however inconsistent evidence supporting the alleged improvements, posterior occlusal contacts being one of the decisive factors. We hypothesized that the distribution of abutment teeth in RPDs influences masticatory performance and functional parameters. To evaluate the masticatory performance and functional parameters in patients with a RPD using a single mathematical parameter (tilting index [TI]) for both jaws that predicts biomechanical behavior on the basis of the distribution of abutment teeth.

**Materials and Methods:**

Masticatory performance was measured in patients wearing long‐time adapted RPDs using the standardized test food optocal, yielding the mean particle size (*X*_50_). Mastication on the preferred and nonpreferred chewing sides was analyzed. Total muscle work (TMW) was calculated using bipolar electromyographic recordings of the masseter and anterior temporalis muscle. Functional parameters were subjected to multiple linear regression analysis including *X*_50_ as a dependent variable and functional units (FU), the number of teeth, bite forces, and sagittal and frontal components of TI (TI *α* and TI *β*) as independent variables.

**Results:**

When the preferred chewing side was tested, none of the investigated parameters correlated significantly with *X*_50_. In contrast, chewing on the nonpreferred side was correlated significantly with performance for most variables (*p* < .05). This means that increased dental support improved chewing performance with RPDs under these conditions.

**Conclusions:**

In well‐adapted RPDs, the distribution of abutment teeth as expressed by the tilting index seems to be of subordinate importance for masticatory performance.

## 1 INTRODUCTION

With a rapid increase in life expectancy, the number of partially dentate patients is also growing. In particular, caries and periodontal diseases lead to tooth loss and/or shortened dental arches. Depending on the number and location of lost teeth, removable partial denture (RPD) is used to restore masticatory function, esthetics, and phonetics. Previous studies have shown that the masticatory performance of patients with RPD is less than for fully dentate subjects (Ikebe et al., 2012). Above all, gender, bite force, location of remaining teeth, and the number of functional units (FU) affect the masticatory performance of patients with RPD (Tumrasvin et al., 2006).

Studies on the biomechanical effects of incorporated RPD have furnished divergent results, depending on the measure of performance used and the number of lost teeth. It has been reported that patients with an extremely shortened dental arch have a poor masticatory performance, which can be improved by wearing a removable partial denture (Arce‐Tumbay et al., 2011). In contrast, other studies found that RPD has no effect on masticatory performance if the premolar regions are intact (Ikebe et al., 2011; Peyron et al., 2004).

For patients with unilateral shortened dental arches, the correlation between bite force and masticatory performance is stronger on the dentate side (Tumrasvin et al., 2005), and the greatest correlation between masticatory performance and bite force is observed for the first molar region (Lujan‐Climent et al., 2008). It can generally be stated that the decrease in masticatory performance among elderly patients is mainly caused by tooth loss, and less by reduced bite force and muscle forces (Ikebe et al., 2011; Peyron et al., 2004).

Monitoring of electric muscle activity (EMG) reveals that symmetrical activation of the masticatory muscles improves chewing performance (Garrett et al., 1995) and that the duration of EMG activity during the chewing cycle is inversely proportional to the stability of the dentition (Balkhi et al., 1993). It has, furthermore, been reported that EMG activity (area under the curve, or integral) during chewing of foods of different hardness is not significantly different for elderly people with full dentition and young subjects. This means that the elderly are still able to adapt excellently in the submaximum range of muscle activation, despite the loss of maximum bite‐force capacity (Peyron et al., 2004). Reduced chewing performance of the elderly might be caused by tooth wear, which reduces the biomechanical effectiveness of the teeth (Giannakopoulos et al., 2014).

**Figure 1:**
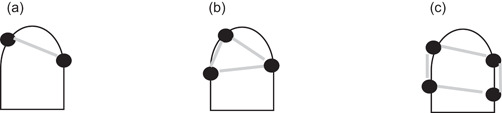
Categories of denture support according to the distribution of abutment teeth. (a) Linear, (b) triangular, and (c) quadrangular

In previous work, biomechanical conditions that affect the chewing performance of partially edentulous patients have been studied by use of classic measures, for example, the “Kennedy” or “Eichner” classification (Ikebe et al., 2010; Rehmann et al., 2015), which characterize linear, triangular, and quadrangular dental support conditions as well as FU (Figure 1).

Essential physiological data for the masticatory system, in particular EMG and bite force measurements, have also been used to determine the neuromuscular effect of tooth loss on the functional behavior of the system. Criteria for evaluation of the biomechanical balance of prostheses on the basis of the arrangement of the residual teeth and other support elements for *both* jaws, by use of a single model, are not available. Such an approach might enable more consistent grading of the biomechanical conditions for prosthetic reconstructions, and, more importantly, better characterization of the biomechanical balance. Additional basic condition in this context represents the adaptability of the neuromuscular system. The target for a realistic assessment of the neuromuscular capacity of the masticatory system is unquestionably the physiology of mastication. Mastication is a complex process involving food breakdown, moistening and dilution in saliva, bolus formation, and swallowing. Masticatory performance was proposed to be the result of two processes: selection of food particles and breakage (Lucas & Luke, 1983). These processes are fundamentally dependent on learned motor patterns, and both should be reflected by the method of testing experimental performance. Commonly, the masticatory performance is investigated in the short term after the incorporation of dentures. However, this experimental design cannot picture the realistic chewing performance after long‐term motor adaptation. Previous investigations substantiated the adaptability of the jaw motor system and have also shown that the motor behavior adapts to new motor tasks by training (Hellmann et al., 2011). This adaptation needs a certain time (Goiato et al., 2010). In this context, it can be supposed that in the fully adapted chewing system the nonpreferred chewing side, which is challenged by unfamiliar chewing, may respond with different performance.

The purpose of this investigation was twofold. First, it correlates the masticatory performance of partially dentate subjects wearing RPD for more than 3 years with two variables, the distribution of individual abutment teeth in the maxilla and mandible, by means of a mathematical model specifically developed to enable estimation of the biomechanical properties of *both* jaws. Second, the gained data on the preferred chewing side were compared with those when patients chewed on the nonpreferred chewing sides. We hypothesized that the distribution of individual abutment teeth in the maxilla and mandible would substantially affect masticatory performance under both conditions. As an additional goal, EMG and bite force was also measured, and classic variables, for example, FU and the number of teeth, were determined to enable an analysis of their correlation with performance on the preferred and nonpreferred chewing sides.

## 2 MATERIALS AND METHODS

### 2.1 Subjects

Twenty‐nine patients (mean age 65.8 ± 8.8 years, 21 female; 8 male) with telescopic RPD participated in the study. The prostheses were incorporated for at least 3 years. Participants underwent a conventional clinical examination used for prosthodontic cases.

The study included 58 jaws and 366 telescopes (166 in the upper and 200 in the lower jaw) distributed with no side preferences. In Table 1 the distribution of the different support conditions is categorized.

**Table 1:** Fifty‐eight jaws and 366 teeth (166 in the upper and 200 in the lower jaw) were distributed with no side preferences under different support conditions

Support	Upper jaw	Lower jaw	Both jaws	Single jaw	Total
Punctual	3	3	1	4	6
Linear	11	12	4	15	23
Triangular	8	6	2	10	14
Quadrangular	1	1	0	2	2
Full dentition	5	6	0	11	11
Complete denture	1	1	0	2	2

*Note*: The number of patients with respective support is depicted.

The study was approved by the Ethics Committee of the University Medical Center, Heidelberg (S‐570/2014) and all patients gave their written consent to the experiments.

### 2.2 Biomechanical model

**Figure 2:**
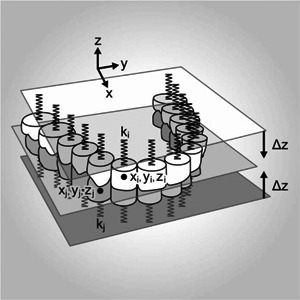
Model for evaluation of the support situation. At the positions of the teeth, springs (stiffness *k*_*i*/*j*_) are attached to a rigid plate in the occlusal plane. Δ*z* denotes the initial deflection of the spring ends toward the occlusal plane necessary to produce a preload

Traditional classification schemes, for example, the “Eichner” or “Kennedy” classification, cannot give a correct indication of mutual support for maxillae and mandibles containing teeth in different positions. For further clarification of this issue, we developed a biomechanical model that quantifies the static equivalence of support conditions in the maxilla and mandible. The occlusal plane (OP) is modeled by a rigid plate. The teeth are modeled by springs that are attached perpendicular to the OP (index *i* was used for all parameters associated with the maxilla whereas index *j* indicated an association with the mandible) at their respective positions (including missing teeth). For mathematical analysis, a Cartesian coordinate system is chosen, which lies in the OP with *x* and *z* pointing in the anterior and vertical directions, respectively (Figure 2).

**Figure 3:**
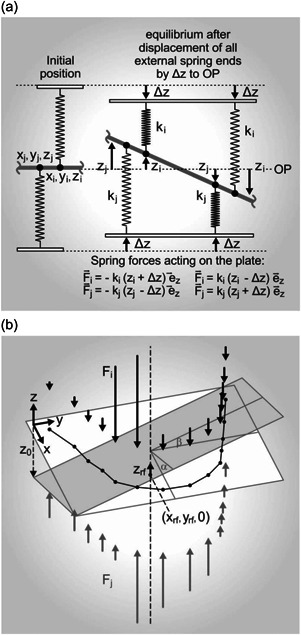
(a) A schematic diagram of the correlation between spring forces acting on the rigid plate and displacements in the new state of equilibrium. (b) Spring forces acting on the rigid plate in the new state of equilibrium (example with RPD supported by gingiva except in the positions of abutment teeth 12, 13, and 32, and 31, 41, and 42). The dashed line shows the line of action of the resulting forces in the maxilla and mandible

Ideally, the occlusal plane and the positions of the teeth are determined for each patient individually. Uniform geometry can, however, be used as a simplification. In both cases, the appropriate stiffness of each spring is chosen in accordance with the local support of the respective partial dentures (tooth, implant, and gingiva). In our biomechanical model, we included mechanical support up to the positions of the second molars (each 14 teeth tooth positions for maxilla and mandible). The model can contain any number of theoretical support positions, for example, *n* = 16 if positions of the wisdom teeth should be included.

To evaluate the equivalence of the support present in the maxilla and mandible, the position of the rigid plate is fixed and all spring ends are moved by a distance Δ*z* toward the OP, thus introducing prestress into the system. When the plate is released, it will move to a new position of equilibrium, depending on the support. This position will usually be displaced and twisted compared with the original position. The mathematical basics of the model are summarized in the appendix.

Tilting of the rigid plate (*α*: tilt in the *x*‐direction, *β*: tilt in the *y*‐direction) is appropriate for evaluation of the equivalence of the spatial distribution of the support elements for both jaws. Under the action of spatially equivalent support in the maxilla and mandible, the plate remains in the horizontal position. The more the situations in the maxilla and mandible differ from this state, the larger becomes the tilting, that is, more unfavorable is the static situation of the system. The vertical displacement at the position of the resultant force in each jaw (*z*_rf_) is a measure of total stiffness differences of the supports in the maxilla and mandible and will always be oriented toward the more resilient side.

Clinically, the algebraic signs of the three variables are possibly misleading; absolute values of |*z*_rf_|, |*α*|, and |*β*| should therefore be used. In statistical analysis of the results from this study, however, only the TI, |*α*|, and |*β*|, were analyzed whereas the effect of total stiffness differences (corresponding with *z*_rf_) was not taken into account.

### 2.3 Masticatory performance

After completing a test cycle, the patients performed three different masticatory performance tests. Each test entailed 15 chewing strokes for habitual and unilateral chewing on the right and left sides of the jaw. Habitual chewing was repeated two times. Standardized artificial test food (Optocal) was used (Pocztaruk Rde et al., 2008) and 17 5.6‐mm cubes formed a portion. Optosil, plaster, alginate, vaseline, and toothpaste were blended in a standardized mixing ratio. The hardness and texture of Optocal make it similar to natural test food, but it can also be chewed by patients wearing a prosthesis (Pocztaruk Rde et al., 2008; Slagter et al., 1993) and it has been used in previous studies (Eberhard et al., 2018).

In this context, habitual chewing enabled the identification of the preferred chewing side. To identify the preferred chewing side, three methods were employed. First, total muscle work (TMW) ratios were used. The side that showed greater muscle activity (TMW) was determined to be the preferred chewing side. Similar methods were applied by Yamasaki et al. (2016) and Ratnasari et al. (2011). Second, observation by an examiner was used as an indicator. Finally, the patients were interviewed and their preferred chewing side was documented. Related to this, other studies have used a questionnaire or a visual analog scale to document the subjective preferred chewing side (Diernberger et al., 2008; Rovira‐Lastra et al., 2016).

It is known that EMG activity is greater on the preferred chewing side during deliberate unilateral chewing (Stohler, 1986). Thus, muscle activity for habitual chewing and deliberate unilateral chewing were compared, and the greater activity was chosen. This pattern is reflected in the measurements from all three performance tests; usually, it is most pronounced during habitual chewing. The activity of the masseter muscle was decisive for determining the chewing side, because this muscle is mainly responsible for the power stroke, whereas the temporalis muscle is more important for the coordination of movements and positioning of the mandible. This is also in agreement with Yamasaki et al. (2015), in which masseter activity was used to determine the actual chewing side.

For silicone test food, the standard approach is to determine the weight of the particles retained by sieves and to curve‐fit the cumulative weight by the use of the Rosin–Rammler equation. In the present study, in contrast to classical sieving methods, optical scanning analysis methods for measuring masticatory performance were used (Eberhard et al., 2012). The approximate weight distributions were determined by the use of the Rosin–Rammler equation and by a least‐squares method (Olthoff et al., 1984; Rosin & Rammler, 1933; Slagter et al., 1993), by use of a MatLab tool (MatLab tool “Rosin Rammler diagram v 1.0” by Ivan Brezani, 2010). The Rosin–Rammler function used was:

$$Q^{-}(X)=1–2^{-\left(X/X_{50}\right)b}.$$

In this equation, *Q* represents the volume percentage of the particles with a size smaller than *X*. *X*_50_ is the median particle size or the size of the theoretical sieve through which 50% of the volume of particles can pass. The variable *b* represents the broadness of the size distribution. A high *X*_50_‐value means that the chewing performance is poor. The particle size is overall large. A low value, on the other hand, means that the particles are quite small and the chewing performance is good (Mowlana et al., 1994; Speksnijder et al., 2009; van der Bilt et al., 1993).

### 2.4 Electromyography

The electromyographic activity of the chewing muscles was recorded by use of Ag/AgCl bipolar surface electrodes (Noraxon, Scottsdale, Arizona, USA). After cleaning the skin with alcohol (70%), electrodes were placed on both sides of the middle part of the masseter and on the anterior temporalis. The reference electrode was positioned on the neck over the seventh vertebra. The EMG signals were differentially amplified (MP100, Acquire 3.9.1 software; Biopac, Santa Barbara, CA, USA), recorded at a sampling rate of 1500 Hz, saved on a personal computer, and band‐pass‐filtered (10–700 Hz) off‐line.

### 2.5 Bite force measurement

**Figure 4:**
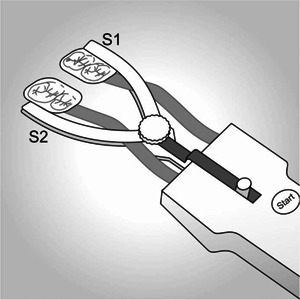
Bitefork with specific characteristics. Bilateral sensors are placed between bite blocks individualized with silicone impression material. S1 and S2: individualized bite blocks

Bite force was measured with a recently available bitefork (BiteFork; ViMeS, Igel, Germany). The two functionally separated sensors of the instrument were placed between prefabricated bite blocks individualized with silicone impression material and positioned between the second premolars and first molars (Figure 4). This configuration enabled simultaneous but separate force recordings for the left and right sides of the jaw. The sampled data were saved on a personal computer for further analysis.

### 2.6 Experimental procedure

After the installation of the EMG recording device, the three masticatory performance tests were conducted in one session. First, the subjects performed three maximum bites in maximum intercuspation. After a test cycle for habitual chewing to familiarize them with the procedure, subjects were asked to chew habitually, followed by chewing on the right side and, finally, on the left side. Habitual chewing was used to identify the preferred chewing side and was repeated two times. Under each condition, 15 chewing cycles were performed. The minced food was spat out and the mouth was rinsed with water to collect all the particles in a filter bag.

The bitefork was adjusted with silicone impression material. Initially, while holding the bitefork in the mouth and stabilizing it by hand, the patient was asked to bite on the bite blocks and to perform some bites to become familiar with the feedback device. If the subject was able to control the bite force by watching a bar on the feedback screen, he/she was asked to bite three times with 50, 100, and 150 N on the device.

### 2.7 Data analysis

The collected and dried chewed artificial test food was scanned and analyzed by use of a validated procedure (Eberhard et al., 2012). *X*_50_ values were calculated by use of the Rosin–Rammler algorithm. The EMG data were analyzed by use of AcqKnowledge 3.9.1 software and a semiautomatic Matlab program. Root mean square (RMS) normalized recordings adjusted to maximum biting EMG were used to compute the area under the curve (integral) of the EMG bursts. TMW for all bilaterally measured muscles was summed for the 15 chewing cycles and analyzed. The ratios of side‐specific TMW (SMW) for the preferred and nonpreferred chewing sides were also analyzed. Bite force recordings were evaluated separately for each side of the jaw.

The tilting indices TI *α* and TI *β* categorize the overall biomechanical balance of the jaws for the respective RPD‐reconstructed dentition. The number of remaining teeth was also counted, and documented as totals for the preferred and nonpreferred chewing sides for each subject. In the same way, FU (antagonistically contacting teeth) for each person were matched with the chewing sides.

To achieve a realistic comparison of the functional data with the remaining dentition, all analyzed recordings were adjusted in relation to the preferred or nonpreferred chewing side (e.g., the EMG data for left‐side chewers were interchanged in comparison with those for the right‐side chewers). Means and standard deviations (SD) were calculated for all results.

### 2.8 Statistics

Spearman correlation was used to analyze correlations between the variables *X*_50_, TI, TMW, FU, bite forces, and the number of teeth. Multiple linear regression analysis was also used. Preferred and nonpreferred chewing sides were analyzed separately. Chewing side differences for TMW, SMW ratios, and bite forces were tested by repeated‐measures analysis of variance. The level of significance was set at *p* < .05.

### 2.9 Ethical approval

All procedures performed in studies involving human participants were in accordance with the ethical standards of the institutional review board and with the 1964 Helsinki declaration and its later amendments.

## 3 RESULTS

**Figure 5:**
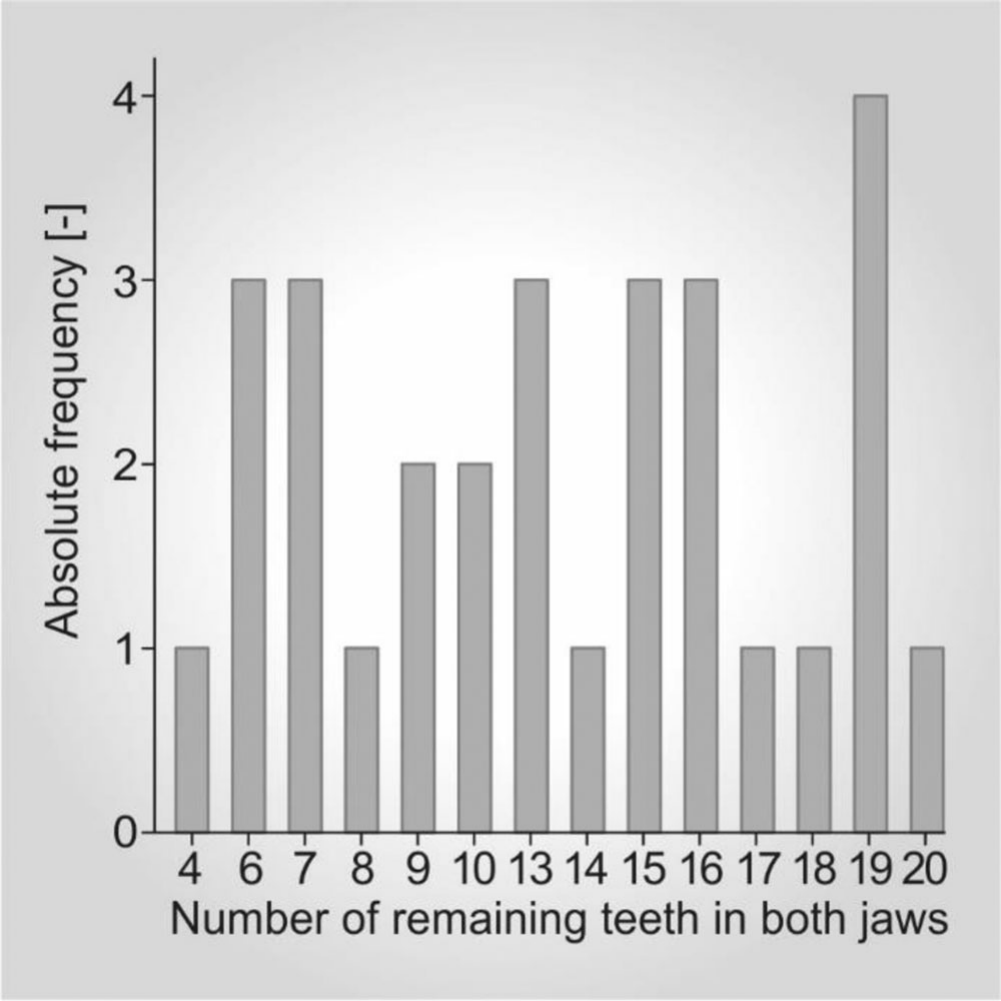
Histogram showing the frequency of the numbers of teeth remaining for all the participants

A broad distribution of the total number of remaining teeth was observed for the maxillae and mandibles; the minimum was three and the maximum 20 (Figure 5). The findings revealed that 22 of the subjects chewed preferentially on the right and seven on the left. The values obtained for TI *α* and TI *β* are listed in Table 2; high and low values represent the most unfavorable and favorable cases, respectively. FU could be detected for 16 subjects but were absent in 13.

**Table 2:** TI *α* and TI *β* (*n* = 29)

TI	Min.	Max.	Mean	SD
*α*	0.03	2.21	0.83	0.59
*β*	0.00	1.30	0.50	0.40

Abbreviation: TI, tilting indices.

The *X*_50_ values presented in Table 3 ranged from 0.50 to 4.82 mm; high scores are indicative of relatively poor and low scores for relatively good performance, respectively. The *X*_50_ values for the nonpreferred chewing side were smaller than those for the preferred side but did not differ significantly (*p* = .68). Values of *X*_50_ and the tilting index β (tilting around a sagittal axis) were significantly positively correlated (*r* = .36, *p* = .05) for chewing on the nonpreferred side (Table 4). No significant correlation (*r* = −0,04, *p* = .85) with TI β was observed for the preferred chewing side, however. Correlations between *X*_50_ and TI *α* values of both preferred and nonpreferred chewing sides were not significant (*r* = .17, *p* = .39 and *r* = .23, *p* = .51, respectively) (Table 4).

**Table 3:** *X*_50_‐values for preferred and nonpreferred chewing sides, *n* = 29 (in mm)

	Min.	Max.	Mean	SD
Preferred	0.5	4.82	2.96	1.11
Nonpreferred	0.87	4.76	3.03	1.11

**Table 4:** Correlations of X_50_ for preferred and nonpreferred chewing side

	TI *α*		TI *β*		Total number of teeth		Number of functional units		Total muscle work	
	*r*	*p*	*r*	*p*	*r*	*p*	*r*	*p*	*r*	*p*
*X*_50_ preferred side	.17	.39	−.04	.85	−.42	.03	−.47	.01	−.37	.05
*X*_50_ nonpreferred side	.23	.51	.36	.05	−.37	.05	−.09	.61	−.33	.08

On the preferred chewing side, the mean number of teeth was 6.52 (SD = 2.6); on the nonpreferred side the mean was 6.10 (SD = 2.7). A significant negative correlation was observed for the total number of teeth and the *X*_50_ values for both the preferred and nonpreferred chewing sides (*r* = −.42, *p* = .03 and *r* = −.37, *p* = .05).

A significant negative correlation was observed between TMW and *X*_50_ for the preferred chewing side (*r* = −.37, *p* = .05), but not for the nonpreferred chewing side (*r* = −.33, *p* = .08, Table 4). TMW values (integrals) for both conditions (Table 5) were not significantly different, however (*p* = .24). The SMW ratios for the preferred (mean = 1.5, SD = 0.3) and nonpreferred (mean = 1.1, SD = 0.3) chewing sides differed significantly (*p* < .0001).

**Table 5:** Total muscle work (TMW, mVs) for preferred and nonpreferred chewing sides, *n* = 29

	Min.	Max.	Mean	SD
Preferred	0.05	0.21	0.1	0.03
Nonpreferred	0.04	0.13	0.08	0.03

None of the bite forces for the preferred and nonpreferred chewing sides correlated significantly with the *X*_50_ values (*p* > .05), and no significant differences between bite forces for either side were found for any force level (Table 6).

**Table 6:** Bite force for preferred and nonpreferred chewing sides at 50, 100, and 150 N, *n* = 29

Bite force (N)	Chewing side	Mean	SD
50	Preferred	23.84	11.50
	Nonpreferred	23.02	11.39
100	Preferred	45.37	21.54
	Nonpreferred	48.39	20.78
150	Preferred	71.16	33.15
	Nonpreferred	67.00	33.74

Multiple linear regression analysis for the preferred chewing side revealed no significant contribution of any independent variable to the variability of the dependent variable *X*_50_. For the nonpreferred chewing side, in contrast, TI *α*, TI *β*, TMW, and FU, in particular, explained 33.3% of the variability, with significant contributions (*p* < .05) (Table 7).

**Table 7:** Multiple linear regression analysis for preferred side (PS) and nonpreferred side (NPS)

	Independent variables	Regression coefficient	SE	Sig.	CI lower	CI upper	*R* ^2^
Preferred side	Constant	3.19	1.21	0.02	0.67	5.72	.15
	Bite force	.015	0.011	0.18	−0.01	0.04	
	TI *α*	.29	0.37	0.44	−0.48	1.06	
	TI *β*	−.16	0.57	0.78	−1.35	1.03	
	Functional units (PS)	−.41	0.21	0.06	−0.85	0.02	
	Number of teeth (PS)	.01	0.11	0.97	−0.22	0.23	
Nonpreferred side	Constant	3.51	0.96	0.001	1.53	5.50	.33
	Bite force	−.01	0.01	0.41	−0.03	0.01	
	TI *α*	.85	0.34	0.02	0.15	1.54	
	TI *β*	1.08	0.51	0.04	0.03	2.13	
	Functional units (NPS)	.39	0.18	0.04	0.01	0.76	
	Number of teeth (NPS)	−.28	0.10	0.01	−0.49	−0.06	

## 4 DISCUSSION

The objective of this study was to quantify the functional effects of different abutment teeth distributions for a sample of patients restored with RPDs that had been worn for at least 3 years. The performance of the nonpreferred chewing side was assumed to correspond to a nonadapted situation, for instance, a condition immediately after tooth loss or a short‐term response after modifying static tooth distribution in the context of various prosthetic reconstruction techniques. In the context of this study, we refer to adaptation in a general sense as the reaction of the organism to an alteration of internal or environmental conditions. Several physiological variables were included in the statistical analysis. For the preferred chewing side, the main result of this investigation is that—on the basis of the rather small data set of this pilot study—masticatory performance correlates neither with a static biomechanical balance of the restored chewing system, characterized by the TI nor with the number of FU, the number of teeth, TMW, or bite force. A small, nonsignificant influence on the number of functional units can be seen on the preferred chewing side as well. We assume that in a larger patient collective, this effect might be more pronounced. Even so, for long‐term adapted RPD and the preferred chewing side static biomechanical variables seem to be of subordinate significance for performance.

In contrast, if the nonpreferred chewing side is used by the patients, the static balance of restorations, characterized by the TI, the FU, and the number of teeth, correlate significantly with *X*_50_ values. The TI *α*, representing the tilting of the denture in the sagittal plane, is of greater importance than the TI *β*, referring to the frontal plane. Summed up, the distribution of abutment teeth has a significant effect on the nonpreferred side. We would attribute this effect to the unfamiliarity of this condition to the patient. The lower TMW indicates that patients may have exerted greater caution when chewing on the nonprefered side. As their masticatory performance tends to increase over an adaptation period (Giannakopoulos et al., 2017), we assume that in an unfamiliar situation, the influence of the distribution of abutment teeth is higher in proportion to overriding factors and thus produces a significant result. It can be argued, whether this unfamiliar situation is generalizable to nonadapted states as described above (e.g., new dentures). To validate this claim, longitudinal studies involving a change in the number of abutment teeth would have to be performed.

On the basis of our findings, the initially stated hypothesis that specific distributions of abutment teeth of RPDs in the maxilla and mandible significantly affect masticatory performance must be rejected for the adapted restored jaw.

The results do, however, support the notion that for adapted neuromuscular systems motor control strategies are adjusted in such a way as to enable them to perform with the best available biomechanical effectiveness. This may imply that, in the long term, the masticatory system does not correspond to static loading conditions in intercuspation, as is generally assumed.

This is also supported by the findings that the ratios of SMW (i.e., working side vs. balancing side; 1.5 for chewing on the preferred side vs. 1.1 for chewing on the nonpreferred side) differ significantly under both chewing conditions; this was not observed for the TMW values. The ratios for the preferred side correspond well to those for natural dentition during unilateral chewing (Proschel & Morneburg, 2010). In the context of our study, it must be considered that during chewing the food bolus is placed unilaterally between the opposing jaws, resulting in bite forces substantially different from those of symmetrical static loading of the dentition or prosthetic reconstruction. Thus, our results challenge a mechanistic approach that is still commonly used for the prediction of the denture dynamics of reconstructions.

Several limitations of this study must be considered. First, the study sample was relatively small and the genders were unequally distributed (this issue should be considered in future investigations). The broad range of abutment teeth distribution may, however, have balanced this possible limitation. Second, all patients were restored with telescopic RPD, which usually ensures a better balance of the denture than the use of clasps. This has consequences for the generalizability of our results. One significant open question also remains unanswered—the overwhelming dominance of preferred right side chewing. The finding is in agreement with population‐based studies (Diernberger et al., 2008) but cannot be explained for our sample on the basis of varying biomechanical conditions because no significant differences between either number of teeth or FU were observed for the preferred and nonpreferred chewing sides. Intraorally, there were no signs of laterality. Other aspects of laterality, such as handedness or eye or ear preference, were not examined. However, studies have shown that there is only a weak positive relationship between the preferred chewing side and other signs of laterality (handedness, eye, ear) (Barcellos et al., 2012). Obviously, simple biomechanical principles seem not to explain this problem. It might be speculated, however, that previous neuromuscular engrams, controlling the complex chewing process, are essential basics for neuromuscular control of this unconscious decision‐making.

It might be argued that the TI, based on the static behavior of the combined jaws, may not be of substantial benefit for estimating the real dynamic conditions in function. However, the results for chewing on the nonpreferred side show that the parameter is sensitive enough to model an effect. Under these conditions the *X*_50_ values are significantly affected by tooth distribution; they also specify an unfavorable distribution as a tilt around the *x*‐axis (angle *β*). The clinical correlate to this observation would be, for example, the common case of the unfavorable dynamics of free‐end saddles (Preston, 2007). The adapted long‐term biomechanical behavior is not predictable by the use of any of the functional variables analyzed, however.

As outlined above, our new biomechanical model describes the static equivalence of both jaw situations in maximum intercuspation and uses the tilt after loading of the combined system as a measure of inequality. A biomechanical description with the help of a continuous mathematical parameter, comprising both jaws, has not previously been available. It is the first attempt to use the distribution of teeth in both jaws as a predictor of the biomechanical balance of the compromised masticatory system. The fact that there are no significant results when the patients were chewing on their habitual chewing side suggests that static considerations that are based on the Eichner‐ or Kennedy‐Classification should be re‐evaluated. When the chewing process is viewed as an asymmetrical movement, it is obvious that a complex procedure such as stabilizing a denture cannot be described with simple static designs. Many treatment plans still follow the idea that only a symmetrical chewing pattern results in good chewing performance. This concept has to be refuted: The chewing process can be regarded as an interaction between acquired neuromuscular patterns, the innate preference for one chewing side, and external conditions. For the first phase of usage after the incorporation of RPD the model might enable the prediction of the quality of performance of the masticatory system and assist decision‐making, regarding the use of additional tooth and/or implant support for a prosthesis. Future investigations must, however, replicate the results of this investigation and validate this new model for cases analyzed before and immediately after the prosthetic restoration of patients. When planning a new restoration, the patient's tooth status could be entered into a software mask based on our mathematical model. The resulting tilting indices show what kind of tilt and torsion can be expected for the planned denture or prosthesis immediately after incorporation. Based on these results, the dentist could decide to add another implant or abutment tooth to achieve a smaller tilting index and greater stability. A major advantage of the developed model is that the tilting index can be calculated individually for each patient time‐saving and efficient.

On the basis of this pilot study, and despite its inherent limitations, it might also be of future interest to evaluate the previously preferred chewing side of patients, to enable comparison of this preference with the preferred chewing side in long‐term use, in particular, because for the human population and all age groups chewing side preference seems to be normal behavior (Barcellos et al., 2012; Nayak et al., 2016; Nissan et al., 2004). For future planning of RPD, this information might be of benefit for obtaining recommendations for placing additional load‐bearing structures (i.e., implants) in the jaws for immediate optimum chewing performance or in patients with a known history of bruxism, to better distribute the generated pathophysiological forces. However, prospective studies have to prove these conceptual hypotheses.

## AUTHOR CONTRIBUTIONS

Lydia Eberhard, Stefan Rues, Jürgen Lenz and Hans J. Schindler contributed to study design. Lea Bach and Lydia Eberhard contributed to data acquisition. Lydia Eberhard, Lea Bach, Stefan Rues and Hans J. Schindler contributed to data analysis. All authors contributed to the drafting of the manuscript and approved the final version.

## CONFLICTS OF INTEREST

The authors declare no conflicts of interest.

## INFORMED CONSENT

Informed consent was obtained from all individual participants included in the study.

## Data Availability

Supporting Information, such as raw data, is available from the corresponding author upon reasonable request.

## 1

The spring forces acting on the rigid plate are given by the expressions:

$$\text{for the maxilla}:\;F_{z,i}={-k}_{i}\left(z_{i}+∆z\right)={-f}_{i}k_{\mathrm{tooth}}\left(z_{i}+∆z\right)$$

$$\text{and for the mandible}:\;F_{z,j}=-k_{j}\left(z_{j}-∆z\right)=-f_{j}k_{\mathrm{tooth}}\left(z_{j}-∆z\right),$$

where *k*_*i*/*j*_ denote spring stiffness and *z*_*i*/*j*_ the displacements from their respective rest positions of the spring ends fixed to the plate. By introducing a reference stiffness, here the stiffness *k*_tooth_ of a tooth, all spring constants *k*_*i*/*j*_ can be specified as values *f*_*i*/*j*_ proportional to this reference stiffness.

In the new state of equilibrium (Figure 3a), the components of all forces and moments acting on the plate must sum to zero:

$$\sum F_{x}=0\;\text{is automatically fulfilled}$$

$$\sum F_{y}=0\;\text{is automatically fulfilled}$$

$$\sum F_{z}=0=\sum_{i=1}^{14}F_{z,i}+\sum_{j=1}^{14}F_{z,j}$$

$$\sum M_{x}=0=\sum_{i=1}^{14}{y_{i}F}_{z,i}+\sum_{j=1}^{14}y_{j}F_{z,j}$$

$$\sum M_{y}=0=\sum_{i=1}^{14}{x_{i}F}_{z,i}+\sum_{j=1}^{14}x_{j}F_{z,j}$$

$$\sum M_{z}=0\;\text{is automatically fulfilled}.$$

All contact points of the springs are located on the plate, that is, in a plane, displaced and tilted into a new equilibrium position relative to the initial position. This new state of equilibrium is characterized by three variables:

- the displacement, *z*_0_, of the rigid plate at the origin, 0, of the coordinate system,
- the gradient *m*_*x*_ of the plate in the *x*‐direction,
- the gradient *m*_*y*_ of the plate in the *y*‐direction.

All deflections must therefore fulfill the side condition:

$${z_{i/j}=z_{0}+m_{x}x}_{i/j}+{m_{y}y}_{i/j}.$$

Thus, the system of equations can be rewritten in the form:

$$\left[\begin{array}{lll} A_{11} & \text{⋯} & A_{13}\\ \text{⋮} & \text{⋱} & \text{⋮}\\ A_{31} & \text{⋯} & A_{33} \end{array}\right]\left[\begin{array}{l} z_{0}/∆z\\ m_{x}/∆z\\ m_{y}/∆z \end{array}\right]=\left[\begin{array}{l} -\sum_{i=1}^{14}f_{i}\;+\sum_{j=1}^{14}f_{j}\;\\ -\sum_{i=1}^{14}y_{i}f_{i}+\sum_{j=1}^{14}y_{j}f_{j}\;\\ -\sum_{i=1}^{14}x_{i}f_{i}+\sum_{j=1}^{14}x_{j}f_{j} \end{array}\right]$$

where:

$$A_{11}=\sum_{i=1}^{14}f_{i}+\sum_{j=1}^{14}f_{j}A_{12}=\sum_{i=1}^{14}f_{i}x_{i}+\sum_{j=1}^{14}{f_{j}x}_{j}\;\;A_{13}=\sum_{i=1}^{14}f_{i}y_{i}+\sum_{j=1}^{14}{f_{j}y}_{j}$$

$$A_{21}=\sum_{i=1}^{14}{y_{i}f}_{i}+\sum_{j=1}^{14}y_{j}f_{j}\;\;\;A_{22}=\sum_{i=1}^{14}{y_{i}f}_{i}x_{i}+\sum_{j=1}^{14}{{y_{j}f}_{j}x}_{j}\;\;A_{23}=\sum_{i=1}^{14}y_{i}f_{i}y_{i}+\sum_{j=1}^{14}{{y_{j}f}_{j}y}_{j}$$

$$A_{31}=\sum_{i=1}^{14}{x_{i}f}_{i}+\sum_{j=1}^{14}x_{j}f_{j}\;\;\;A_{32}=\sum_{i=1}^{14}{x_{i}f}_{i}x_{i}+\sum_{j=1}^{14}{{x_{j}f}_{j}x}_{j}\;\;A_{33}=\sum_{i=1}^{14}x_{i}f_{i}y_{i}+\sum_{j=1}^{14}{{x_{j}f}_{j}y}_{j}$$

The system of equations shows that *z*_0_, *m*_*x*_, and *m*_*y*_ are proportional to the auxiliary quantity ∆ *z* and can therefore be scaled with ∆ *z*. The behavior of the system does not, furthermore, depend on the reference stiffness *k*_tooth_, but only on the stiffness ratios *f*_*i*/*j*_.

By means of the inverse, *A* ^−1^, of matrix *A* the unknown scaled variables can be calculated rather easily:

$$\left[\begin{array}{l} z_{0}/∆z\\ m_{x}/∆z\\ m_{y}/∆z \end{array}\right]={\underline{A}}^{-1}\;\left[\begin{array}{l} -\sum_{i=1}^{14}f_{i}\;+\sum_{j=1}^{14}f_{j}\;\\ -\sum_{i=1}^{14}y_{i}f_{i}+\sum_{j=1}^{14}y_{j}f_{j}\;\\ -\sum_{i=1}^{14}x_{i}f_{i}+\sum_{j=1}^{14}x_{j}f_{j} \end{array},\;\right].$$

To avoid normalized variables the deflection Δ*z* = 1 mm is chosen in the discussion below.

Because the position of the coordinate system within the occlusal plane can be chosen arbitrarily, the deflection *z*_0_ is not a specifically meaningful mechanical variable. Thus, the translation *z*_rf_ (Figure 3b) at the position of the line of action of the resulting forces (*x*_rf_, *y*_rf_) in the maxilla (which is identical to that in the mandible)

$${z_{\mathrm{rf}}=z_{0}+m_{x}x}_{\mathrm{rf}}+{m_{y}y}_{\mathrm{rf}}$$

is introduced with:

$$x_{\mathrm{rf}}=\sum_{i=1}^{14}f_{i}\left(z_{i}+∆z\right)x_{i}/\sum_{i=1}^{14}f_{i}\left(z_{i}+∆z\right)\;\text{and}$$

$$y_{\mathrm{rf}}=\sum_{i=1}^{14}f_{i}\left(z_{i}+∆z\right)y_{i}/\sum_{i=1}^{14}f_{i}\left(z_{i}+∆z\right).$$

*z*_rf_ is zero if the overall stiffnesses in the maxilla and mandible are equal. If the overall stiffnesses are different, the translation |*z*_rf_| is directed toward the more resilient side.

Furthermore, instead of the use of gradients (*m_x_* and *m_y_*, describing the incline of the new equilibrium position in *x*‐ and *y*‐direction), angles associated with these gradients (*α* and *β*) can be specified:

$\alpha =\tan^{-1}\left[m_{x}\left(∆z=1\;\text{mm}\right)\right]\;(\text{corresponds to rotation around the }y‐\text{axis}),$

$\beta =\tan^{-1}[m_{y}\left(∆z=1\;\text{mm}\right)]\;(\text{corresponds to rotation around the }x‐\text{axis}).$

To summarize, the equilibrium state of the plate (after prestressing with Δz = 1 mm) can be described by use of the set of variables *z*_rf_, *α*, and *β*.

## References

[cre2576-bib-0001] Arce‐Tumbay, J. , Sanchez‐Ayala, A. , Sotto‐Maior, B. S. , Senna, P. M. , & Campanha, N. H. (2011). Mastication in subjects with extremely shortened dental arches rehabilitated with removable partial dentures. The International Journal of Prosthodontics, 24(6), 517–519. 22146249

[cre2576-bib-0002] Balkhi, K. M. , Tallents, R. H. , Katzberg, R. W. , Murphy, W. , & Proskin, H. (1993). Activity of anterior temporalis and masseter muscles during deliberate unilateral mastication. Journal of Orofacial Pain, 7(1), 89–97. 8467303

[cre2576-bib-0003] Barcellos, D. C. , da Silva, M. A. , Batista, G. R. , Pleffken, P. R. , Pucci, C. R. , Borges, A. B. , Rocha Gomes Torres, C. , & Gonçalves, S. E. (2012). Absence or weak correlation between chewing side preference and lateralities in primary, mixed and permanent dentition. Archives of Oral Biology, 57(8), 1086–1092. 22469391 10.1016/j.archoralbio.2012.02.022

[cre2576-bib-0004] van der Bilt, A. , Abbink, J. H. , Mowlana, F. , & Heath, M. R. (1993). A comparison between data analysis methods concerning particle size distributions obtained by mastication in man. Archives of Oral Biology, 38(2), 163–167. 8476346 10.1016/0003-9969(93)90202-w

[cre2576-bib-0005] Diernberger, S. , Bernhardt, O. , Schwahn, C. , & Kordass, B. (2008). Self‐reported chewing side preference and its associations with occlusal, temporomandibular and prosthodontic factors: Results from the population‐based Study of Health in Pomerania (SHIP‐0). Journal of Oral Rehabilitation, 35(8), 613–620. 18699970 10.1111/j.1365-2842.2007.01790.x

[cre2576-bib-0006] Eberhard, L. , Oh, K. , Eiffler, C. , Rammelsberg, P. , Kappel, S. , Schindler, H. J. , & Giannakopoulos, N. N. (2018). Adaptation to new complete dentures‐is the neuromuscular system outcome‐oriented or effort‐oriented? Clinical Oral Investigations, 22(6), 2309–2317. 29484547 10.1007/s00784-017-2331-8

[cre2576-bib-0007] Eberhard, L. , Schindler, H. J. , Hellmann, D. , Schmitter, M. , Rammelsberg, P. , & Giannakopoulos, N. N. (2012). Comparison of particle‐size distributions determined by optical scanning and by sieving in the assessment of masticatory performance. Journal of Oral Rehabilitation, 39(5), 338–348. 22220913 10.1111/j.1365-2842.2011.02275.x

[cre2576-bib-0008] Garrett, N. R. , Kaurich, M. , Perez, P. , & Kapur, K. K. (1995). Masseter muscle activity in denture wearers with superior and poor masticatory performance. Journal of Prosthetic Dentistry, 74(6), 628–636. 8778388 10.1016/s0022-3913(05)80316-6

[cre2576-bib-0009] Giannakopoulos, N. N. , Corteville, F. , Kappel, S. , Rammelsberg, P. , Schindler, H. J. , & Eberhard, L. (2017). Functional adaptation of the masticatory system to implant‐supported mandibular overdentures. Clinical Oral Implants Research, 28(5), 529–534. 27001374 10.1111/clr.12830

[cre2576-bib-0010] Giannakopoulos, N. N. , Wirth, A. , Braun, S. , Eberhard, L. , Schindler, H. J. , & Hellmann, D. (2014). Effect of the occlusal profile on the masticatory performance of healthy dentate subjects. The International Journal of Prosthodontics, 27(4), 383–389. 25010884 10.11607/ijp.3793

[cre2576-bib-0011] Goiato, M. C. , Garcia, A. R. , Dos Santos, D. M. , & Zuim, P. R. (2010). Analysis of masticatory cycle efficiency in complete denture wearers. Journal of Prosthodontics, 19(1), 10–13. 19765198 10.1111/j.1532-849X.2009.00520.x

[cre2576-bib-0012] Hellmann, D. , Giannakopoulos, N. N. , Blaser, R. , Eberhard, L. , Rues, S. , & Schindler, H. J. (2011). Long‐term training effects on masticatory muscles. Journal of Oral Rehabilitation, 38(12), 912–920. 21569075 10.1111/j.1365-2842.2011.02227.x

[cre2576-bib-0013] Ikebe, K. , Matsuda, K. , Kagawa, R. , Enoki, K. , Okada, T. , Yoshida, M. , & Maeda, Y. (2012). Masticatory performance in older subjects with varying degrees of tooth loss. Journal of Dentistry, 40(1), 71–76. 22037296 10.1016/j.jdent.2011.10.007

[cre2576-bib-0014] Ikebe, K. , Matsuda, K. , Kagawa, R. , Enoki, K. , Yoshida, M. , Maeda, Y. , & Nokubi, T. (2011). Association of masticatory performance with age, gender, number of teeth, occlusal force and salivary flow in Japanese older adults: Is ageing a risk factor for masticatory dysfunction? Archives of Oral Biology, 56(10), 991–996. 21529776 10.1016/j.archoralbio.2011.03.019

[cre2576-bib-0015] Ikebe, K. , Matsuda, K. , Murai, S. , Maeda, Y. , & Nokubi, T. (2010). Validation of the Eichner index in relation to occlusal force and masticatory performance. The International Journal of Prosthodontics, 23(6), 521–524. 21209986

[cre2576-bib-0016] Lucas, P. W. , & Luke, D. A. (1983). Methods for analysing the breakdown of food in human mastication. Archives of Oral Biology, 28(9), 813–819. 6579911 10.1016/0003-9969(83)90037-7

[cre2576-bib-0017] Lujan‐Climent, M. , Martinez‐Gomis, J. , Palau, S. , Ayuso‐Montero, R. , Salsench, J. , & Peraire, M. (2008). Influence of static and dynamic occlusal characteristics and muscle force on masticatory performance in dentate adults. European Journal of Oral Sciences, 116(3), 229–236. 18471241 10.1111/j.1600-0722.2008.00530.x

[cre2576-bib-0018] Mowlana, F. , Heath, M. R. , Van der Bilt, A. , & Van der Glas, H. W. (1994). Assessment of chewing efficiency: A comparison of particle size distribution determined using optical scanning and sieving of almonds. Journal of Oral Rehabilitation, 21(5), 545–551. 7996338 10.1111/j.1365-2842.1994.tb01168.x

[cre2576-bib-0019] Nayak, U. A. , Sharma, R. , Kashyap, N. , Prajapati, D. , Kappadi, D. , Wadhwa, S. , Gandotra, S. , & Yadav, P. (2016). Association between chewing side preference and dental caries among deciduous, mixed and permanent dentition. Journal of Clinical and Diagnostic Research, 10(9), 5–8. 10.7860/JCDR/2016/20620.8422 PMC5072069 27790569

[cre2576-bib-0020] Nissan, J. , Gross, M. D. , Shifman, A. , Tzadok, L. , & Assif, D. (2004). Chewing side preference as a type of hemispheric laterality. Journal of Oral Rehabilitation, 31(5), 412–416. 15140165 10.1111/j.1365-2842.2004.01256.x

[cre2576-bib-0021] Olthoff, L. W. , van der Bilt, A. , Bosman, F. , & Kleizen, H. H. (1984). Distribution of particle sizes in food comminuted by human mastication. Archives of Oral Biology, 29(11), 899–903. 6596036 10.1016/0003-9969(84)90089-x

[cre2576-bib-0022] Peyron, M. A. , Blanc, O. , Lund, J. P. , & Woda, A. (2004). Influence of age on adaptability of human mastication. Journal of Neurophysiology, 92(2), 773–779. 15277595 10.1152/jn.01122.2003

[cre2576-bib-0023] Pocztaruk Rde, L. , Frasca, L. C. , Rivaldo, E. G. , Fernandes Ede, L. , & Gaviao, M. B. (2008). Protocol for production of a chewable material for masticatory function tests (Optocal ‐ Brazilian version). Brazilian Oral Research, 22(4), 305–310. 19148384 10.1590/s1806-83242008000400004

[cre2576-bib-0024] Preston, K. P. (2007). The bilateral distal extension removable partial denture: Mechanical problems and solutions. The European Journal of Prosthodontics and Restorative Dentistry, 15(3), 115–121. 17970318

[cre2576-bib-0025] Proschel, P. A. , & Morneburg, T. R. (2010). Indications for jaw gape‐related control of relative muscle activation in sequent chewing strokes. Journal of Oral Rehabilitation, 37(3), 178–184. 19968765 10.1111/j.1365-2842.2009.02036.x

[cre2576-bib-0026] Ratnasari, A. , Hasegawa, K. , Oki, K. , Kawakami, S. , Yanagi, Y. , Asaumi, J. I. , & Minagi, S. (2011). Manifestation of preferred chewing side for hard food on TMJ disc displacement side. Journal of Oral Rehabilitation, 38(1), 12–17. 20673297 10.1111/j.1365-2842.2010.02128.x

[cre2576-bib-0027] Rehmann, P. , Podhorsky, A. , & Wostmann, B. (2015). Treatment outcomes of cantilever fixed partial dentures on vital abutment teeth: A retrospective analysis. The International Journal of Prosthodontics, 28(6), 577–582. 26523715 10.11607/ijp.4114

[cre2576-bib-0028] Rosin, P. , & Rammler, E. (1933). Gesetzmaessigkeiten in der Kornzusammensetzung des Zementes. Zement, 31, 427–433.

[cre2576-bib-0029] Rovira‐Lastra, B. , Flores‐Orozco, E. I. , Ayuso‐Montero, R. , Peraire, M. , & Martinez‐Gomis, J. (2016). Peripheral, functional and postural asymmetries related to the preferred chewing side in adults with natural dentition. Journal of Oral Rehabilitation, 43(4), 279–285. 26549578 10.1111/joor.12369

[cre2576-bib-0030] Slagter, A. P. , Bosman, F. , & Van der Bilt, A. (1993). Comminution of two artificial test foods by dentate and edentulous subjects. Journal of Oral Rehabilitation, 20(2), 159–176. 8468627 10.1111/j.1365-2842.1993.tb01599.x

[cre2576-bib-0031] Speksnijder, C. M. , Abbink, J. H. , van der Glas, H. W. , Janssen, N. G. , & van der Bilt, A. (2009). Mixing ability test compared with a comminution test in persons with normal and compromised masticatory performance. European Journal of Oral Sciences, 117(5), 580–586. 19758256 10.1111/j.1600-0722.2009.00675.x

[cre2576-bib-0032] Stohler, C. S. (1986). A comparative electromyographic and kinesiographic study of deliberate and habitual mastication in man. Archives of Oral Biology, 31(10), 669–678. 3477211 10.1016/0003-9969(86)90096-8

[cre2576-bib-0033] Tumrasvin, W. , Fueki, K. , & Ohyama, T. (2006). Factors associated with masticatory performance in unilateral distal extension removable partial denture patients. Journal of Prosthodontics, 15(1), 25–31. 16433648 10.1111/j.1532-849X.2006.00065.x

[cre2576-bib-0034] Tumrasvin, W. , Fueki, K. , Yanagawa, M. , Asakawa, A. , Yoshimura, M. , & Ohyama, T. (2005). Masticatory function after unilateral distal extension removable partial denture treatment: Intra‐individual comparison with opposite dentulous side. Journal of Medical and Dental Sciences, 52(1), 35–41. 15868739

[cre2576-bib-0035] Yamasaki, Y. , Kuwatsuru, R. , Tsukiyama, Y. , Matsumoto, H. , Oki, K. , & Koyano, K. (2015). Objective assessment of actual chewing side by measurement of bilateral masseter muscle electromyography. Archives of Oral Biology, 60(12), 1756–1762. 26433193 10.1016/j.archoralbio.2015.09.010

[cre2576-bib-0036] Yamasaki, Y. , Kuwatsuru, R. , Tsukiyama, Y. , Oki, K. , & Koyano, K. (2016). Objective assessment of mastication predominance in healthy dentate subjects and patients with unilateral posterior missing teeth. Journal of Oral Rehabilitation, 43(8), 575–582. 27121170 10.1111/joor.12403

